# Mixed-reality-based human-animal interaction can relieve mental stress

**DOI:** 10.3389/fvets.2023.1102937

**Published:** 2023-03-16

**Authors:** Heewon Na, Suh-Yeon Dong

**Affiliations:** Human Computer Interaction Laboratory, Department of Information Technology Engineering, Sookmyung Women's University, Seoul, Republic of Korea

**Keywords:** human-animal interaction, mixed reality, virtual animal, mental healthcare, stress relief

## Abstract

**Introduction:**

Interacting with animals has been demonstrated to possess the healing benefits to humans. However, there are limitations in physical interaction due to COVID-19 and safety issues. Therefore, as an alternative, we created mixed-reality (MR)-based human-animal interaction (HAI) content and experimentally verified its effect on mental stress reduction.

**Methods:**

We created three types of interactive content: observing the movement of a non-reactive virtual cat, interacting with a virtual cat whose responses can be seen, and interacting with a virtual cat whose responses can be both seen and heard. The experiment was performed by 30 healthy young women, and a mental arithmetic task was used to induce mild mental stress before experiencing each content. During the experiment, the subject's electrocardiogram was continuously recorded, and the psychological state was evaluated through a questionnaire.

**Results:**

The results showed that MR-based virtual cat content significantly reduces mental stress and induces positive emotions after stressful situations. In particular, when the virtual cat provided audiovisual feedback, the activation amount of the parasympathetic nervous system and the increase of positive emotions were the greatest.

**Discussion:**

Based on this encouraging research result, this method should be further investigated to see if it can replace real HAI for human mental health management.

## 1. Introduction

Human-animal interactions (HAI) have been studied over the past two decades to assess their therapeutic value. Since there is a strong bond between humans and animals, interactions with animals are widely believed to reduce human anxiety, loneliness, and depression, thereby reduce severity of diseases and relieve stress (1). The Animal Visitation Program (AVP) is a program on nearly 1,000 U.S. university campuses that aims to reduce university students' stress caused by other factors providing them with the opportunity to interact with real animals such as cats and dogs (2). In the previous studies, the AVP has demonstrated its effect of relieving the perceived stress momentarily (3, 4), increasing the level of positive emotions, and decreasing the level of negative emotions (5).

However, one study highlighted that real HAIs can cause animal welfare problems. Some dogs participating in AVP exhibited stress behaviors such as lip licking and yawning (6). So while HAIs may provide healing effects in humans, they may also increase stress in animals. In addition, there are several limitations to supporting HAI in universities, such as allergies to animals, safety issues, and a lack of space. In addition, face-to-face activity has been greatly reduced due to COVID-19, and HAI has become more difficult due to the risk of contagiousness from contact with the same animal. To overcome these limitations, interactions with virtual animals can be considered an alternative to interacting with real ones.

Our previous study first investigated whether a stress-relieving effect could be observed even by replacing a real animal with a virtual one. To this end, the physiological and psychological responses of participants were compared after interaction with a virtual cat and after viewing photos of real cats. Surveys and electrocardiograms (ECGs) showed that interactions with virtual cats can reduce negative emotions and induce positive emotions in users in stressful situations (7). However, it was not clear whether the resulting effect was directly due to the interaction. Since many participants were unfamiliar with the experience in a mixed environment, proposed MR content may have had a greater effect compared to viewing static images. This study aims to understand how the stress-relieving effects of virtual cats are caused by diversifying the interaction types. MR content without interaction was set as a virtual cat moving alone, while MR content with interaction was set as a virtual cat interacting with the user and reacting in various ways according to the user's commands. Moreover, unlike the previous study, the virtual cat's auditory feedback was added for a more realistic interaction, and the virtual cat's reaction in the interaction condition was divided into visual-only and audiovisual. The stress-relieving effect was observed by the survey and heart-rate variability (HRV) from ECGs. HRV analysis specifically focused on the mean HR (Heart Rate; average heart rate). We hypothesize that this approach will lead to a more detailed understanding of which aspects of interactions with virtual animals cause stress-reducing effects.

## 2. Related works

Lin et al. reported that pet games can provide emotional support through interaction with virtual pets, without involving animal-related issues such as allergies (8). It was also noted that interactions with virtual pets can have the effect of promoting collaboration, and empathy for users. Norouzi's study found that walking with an AR dog affects a user's walking speed, passing distance, and head rotation. In particular, it was found that if an AR dog reacted to a collision with another person, it increased the user's co-presence and had a positive effect on the user's behavior (9). Furthermore, some other studies have shown that interactions with virtual animals can improve the academic performance of students and have positive effects on their physical activities. One study designed and built a mixed reality system for children to train and play with virtual pets (10). Interactions with virtual pets have succeeded in motivating the treatment groups for physical activity, demonstrating the real potential of mixed reality to influence motor behavior in children. Another study linked a user's daily steps to the growth of a virtual fish in a fishbowl to encourage more physical activity (11). They also succeeded in improving their physical activity through collaboration and competition with other users. Finally, there was a study that showed that animals play various educational roles in a digital environment, helping children to motivate, reflect, and interact with members (12).

Unlike the existing entertainment platforms, augmented reality (AR) and mixed reality (MR) techniques will allow virtual animals to coexist with humans in the real world. MR glasses allow users to interact with virtual animals in the real world. Additionally, the graphical representation of virtual animals can provide users with more advanced graphics and an appearance that resembles real animals compared to robots and desktops used so far (13). The presence or absence of an animal reaction to the user's actions may also have an effect. In one study, to solve a mathematical fraction problem, an interactive virtual reality learning activity was compared with learning by observing the learning of a robot without interaction. The average number of correct answers was slightly higher for interactive learning activities (14). Also, a user's emotions may vary depending on whether an auditory sound is included. One study demonstrated that the effects of sounds from nature and footsteps induced by user movement have statistically significant effects on presence and the feeling of “being there” (15).

## 3. Program design and implementation

### 3.1. Platform overview

Microsoft HoloLens^1^ is a pair of 3D holographic glasses that can recognize and utilize the surrounding environment through spatial feature point extraction methods with a central processing unit (CPU), graphics processing unit (GPU), and holographic processing unit (HPU) (16). Its superior features over traditional AR devices include a stereoscopic 3D display, gaze control, gesture control, spatial sound development, and spatial mapping (17). In order to create content for HoloLens, it is necessary to use the Universal Windows Platform (UWP). Unity is a platform that supports the UWP and integrates and links component assets used within a project, such as 3D objects, and audio (18). Furthermore, Microsoft recently restructured the Mixed Reality Toolkit (MRTK), a powerful SDK useful for developing applications for HoloLens (19). In this study, MRTK v2.4.0 was used to develop MR content. Virtual animals were animated and controlled within Unity through the use of C# using Visual Studio 2019.

### 3.2. Animations

**Figure 1:**
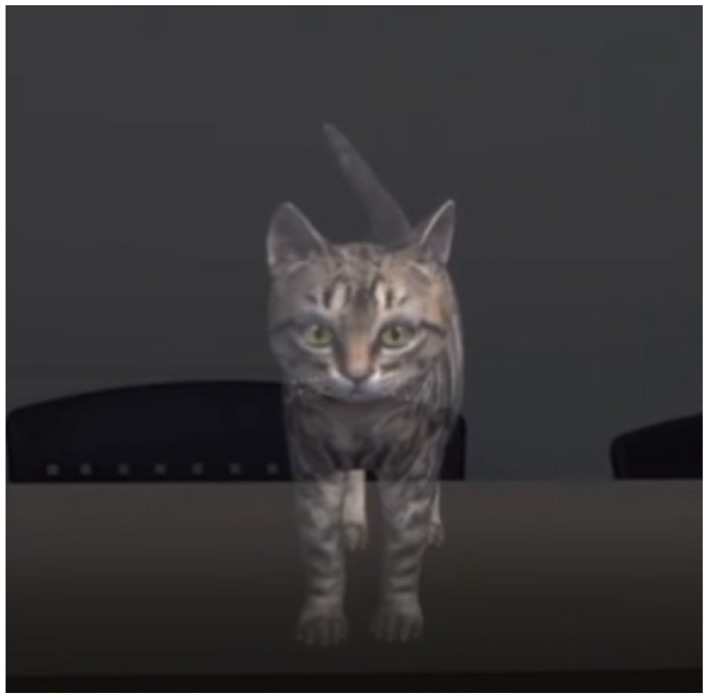
The idle state of virtual cat.

Schwind et al. noted that if the virtual cat model was more natural, of higher quality, and had a less intimidating appearance, users could feel more comfortable with the virtual cat model and interact better with it (20). Considering this aspect, this study used the “Kitten (short)” asset^2^ from the Unity Asset Store. Developers could manipulate the cat's behavior by animating its actions such as sitting, running, and jumping, based on various conditions. In this study, nine behaviors were constructed: idle, eating, walking, jumping, sitting, sleeping, running, lying down, and showing affection. The idle is a default state as shown in Figure 1. For each animation, a bool-type parameter was connected with the idle state that will control the transition. The virtual cat slept when the content started, went idle as soon as the user woke it up, and remained idle whenever the user isn't interacting with it. The parameter value was initialized to false which means to maintain idle. An animation was activated when its parameter becomes true under the user's control. After the animation was finished, its parameter returned to false and the cat's state returned to idle. To ensure that the movement of the virtual cat was not interrupted, transitions connected in the direction of entering an idle state had a fixed end time.

### 3.3. Gestures

**Figure 2:**
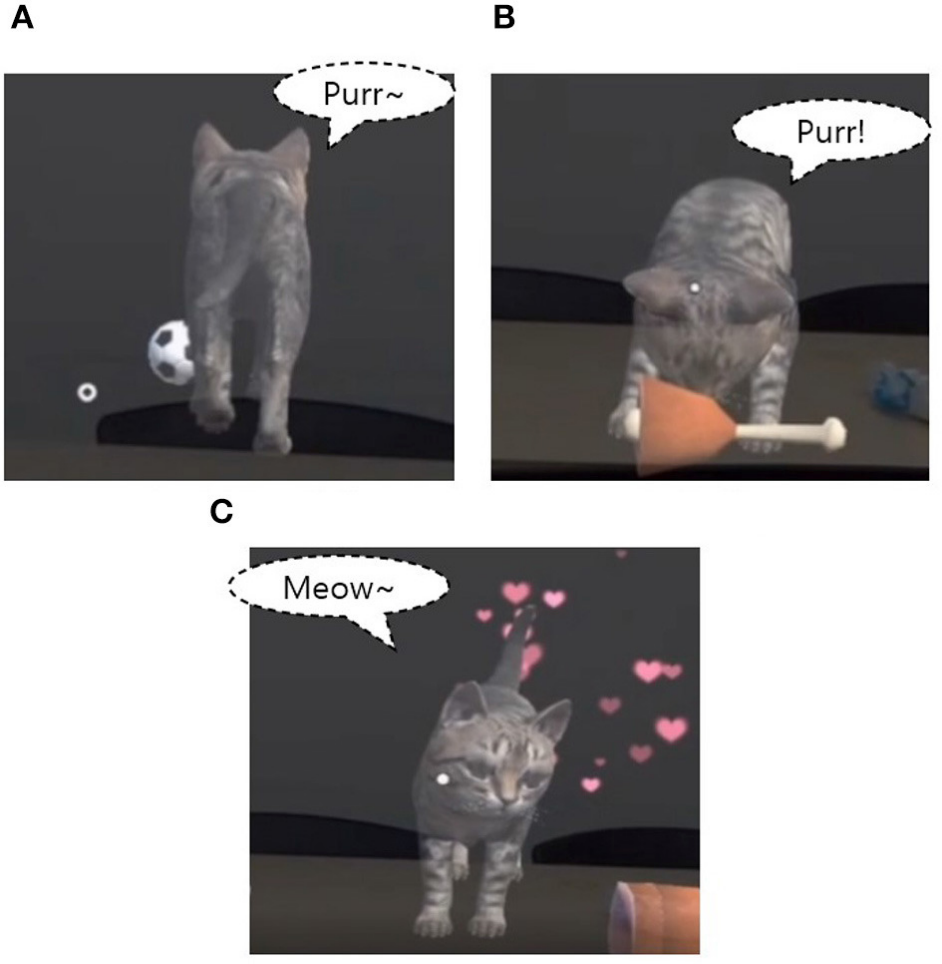
Examples of interaction with gestures. **(A)** Catching a ball, **(B)** feeding, and **(C)** petting.

An MRTK profile for the HoloLens 1 version was created and the input action handler was used to recognize user gestures. In HoloLens, the air-tap is a gesture of gently bending the index finger as if tapping the air while clenching a fist with the back of the user's right hand facing upward. The MRTK input action handler recognizes this gesture as “Select.” Interaction through gestures consisted of catching, feeding, and stroking the ball as shown in Figure 2. Additionally, in the audiovisual condition, cat sounds were added for a more realistic interaction. The sounds of the cat were not added for all gestures or voice commands, but sounds were added only in situations where the cat mainly made sounds by referring to actual cat videos. The cat sounds were selected from Free Cat Sound Effects.^3^

First, as shown in Figure 2A, when the user air-taps the ball, the cat follows the ball, bites it, and brings it back to the user. At this time, the sound of a cat excited while chasing the ball is played. Afterward, when the virtual cat bites the ball, the sound stops and the virtual cat runs to the user. For this animation, we implemented the code in C# to control the position, movement speed, and rotation of the ball and set it to be activated when the air tap is performed. The virtual cat rotates in the direction of the ball as it rolls and a “run” animation is activated to move along the ball's position. When the cat's position reaches the ball, the cat bites the ball and approaches the user's position. Also, when the cat returns to its original position, the ball is deactivated.

Next, when the user air-taps the fish or ham, the virtual cat is set to eat it gradually (Figure 2B). The fish and ham consist of four parts, time is allocated through the IEnumerator, and each part is sequentially deactivated to express the cat's gradual eating of ham and fish. When food is placed in front of the virtual cat, the virtual cat makes a hungry sound and starts eating, and returns to idle again when only the bones of the ham and fish are left. Also, the star-shaped particle effect spreads after the cat has finished eating, indicating that the cat likes the food.

Finally, the user can pet the cat. When the virtual cat is idle, users can air-tap the cat to make it feel good and cute. At this time, to express the cat's satisfaction, a heart-shaped particle effect is added and a cute meow sound is added (Figure 2C).

### 3.4. Voice commands

**Figure 3:**
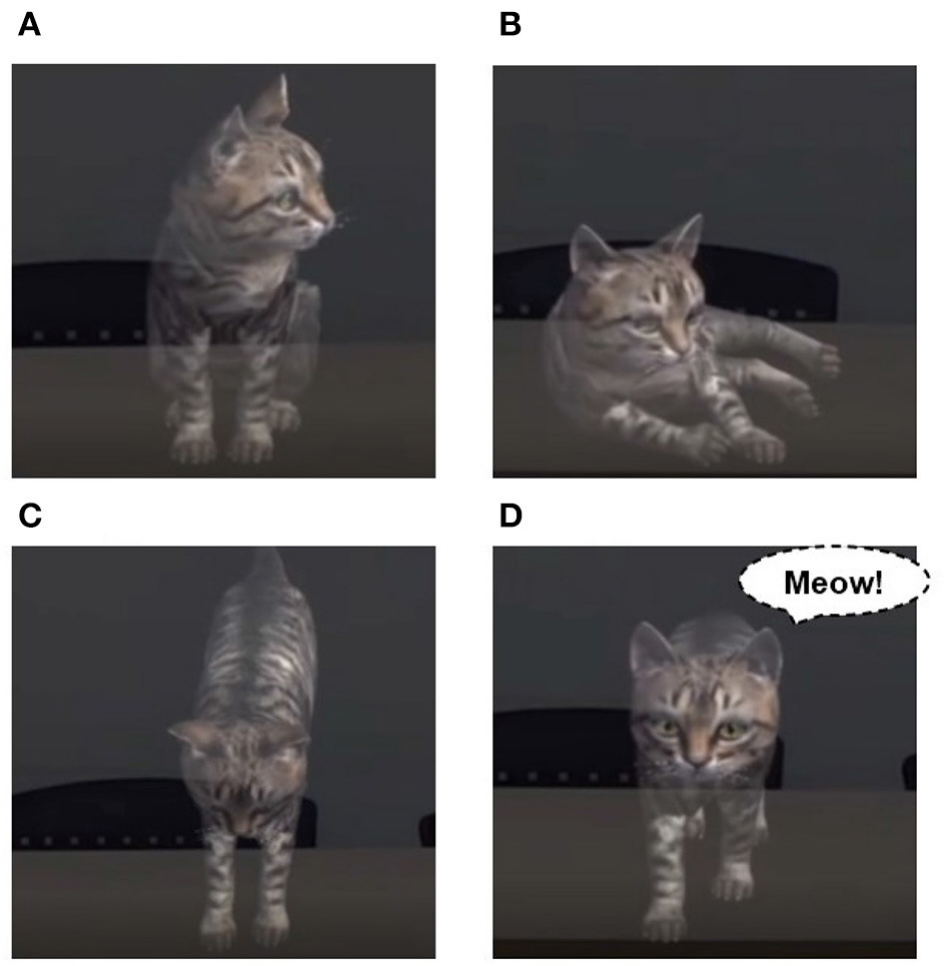
Examples of interaction with voice commands. **(A)** Sit down, **(B)** lie down, **(C)** jump, and **(D)** come here.

The game voice control plugin ^4^ is used to recognize the user's voice. In this study, four types of voice commands and interactions are constructed as shown in Figure 3. GameVoiceControl is loaded into the Hierarchy, English is chosen as a command language, and Commands are registered with the Textlog so the four words “ump,” “Lie down,” “Come here,” and “Sit down” are recognized. A voice command is displayed and recognized in the Text object, and an animation is triggered when it matches one of the four commands. In the case of “Come here,” the cat makes a happy sound as it approaches the user. When the motion is complete, the animation is disabled again and goes back to idle.

## 4. Experiment

### 4.1. Subjects

The experiment involved 30 healthy female adults in their twenties (mean (M) ± standard deviation (SD) age 22.07 ± 2.29). Subjects with a history of heart disease and neuropsychiatric disorders, those currently taking medications, pregnant women, or women about to become pregnant were excluded from the study. Additionally, subjects who normally wear glasses were asked to wear contact lenses in order to use HoloLens during the experiment. All experimental procedures involving human subjects were approved by the affiliated institutional review board (SMWU-2008-HR-073). The entire experimental procedure was introduced orally to the participants and written consent was obtained. All participants who took part in the experiment were given a monetary reward after the experiment was over. In addition, the experiment was conducted 1:1 with the experimenter and the subject in an independent room.

### 4.2. Experimental design

The experiment was conducted in the order of “Preparation—Tutorial—Baseline— Experiment 1—Rest—Experiment 2—Rest—Experiment 3,” and the total time required was approximately 60 min. For convenience, subjects wore the HoloLens only when experiencing Tutorial and MR contents, and took it off for the rest of the experiment. Each phase of the experiments will be explained in detail.

#### 4.2.1. Preparation

At the beginning of the experiment, a preparatory phase gave the subject the details of attaching the ECG sensor to their chest on their own. Also, the first survey was conducted to measure the baseline psychological states of the subjects. The surveys were conducted 7 times in total to measure psychological changes, first time after the Baseline and two times per Experiment phase.

#### 4.2.2. Tutorial

**Figure 4:**
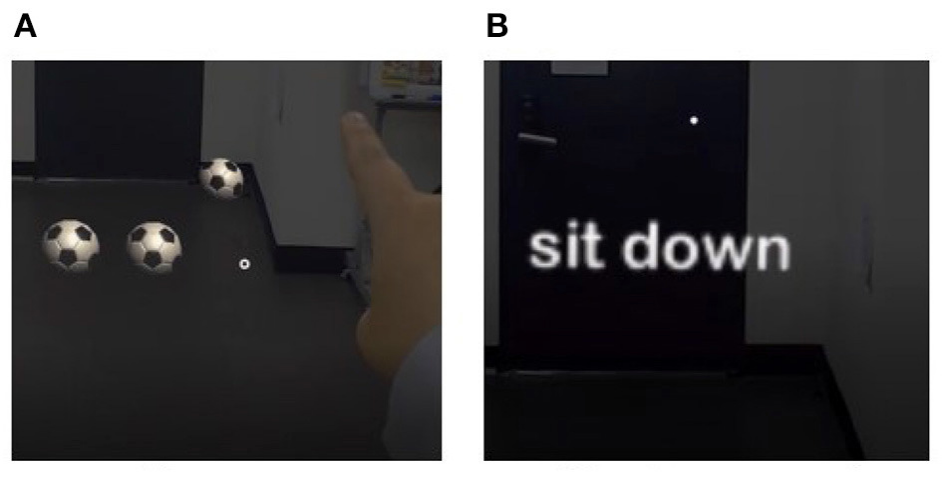
Scene examples of the tutorial phase. **(A)** Gestures and **(B)** voice command.

After wearing the HoloLens, a tutorial was conducted so that the user can become familiar with the MR environment, gestures, and voice commands. As shown in Figure 4, the subject went through an acclimatization process by speaking into a microphone and rolling a ball using the air tap gesture to confirm the operation of the speech recognition system. The tutorial was repeated until the subject determined that they were sufficiently familiar with speech recognition and gestures.

#### 4.2.3. Baseline

The subject was then asked to sit still for 3 min to measure the baseline states by recording ECGs and responding the surveys. The survey was conducted after 3-min ECG recording is completed.

#### 4.2.4. Experiment and rest

**Figure 5:**
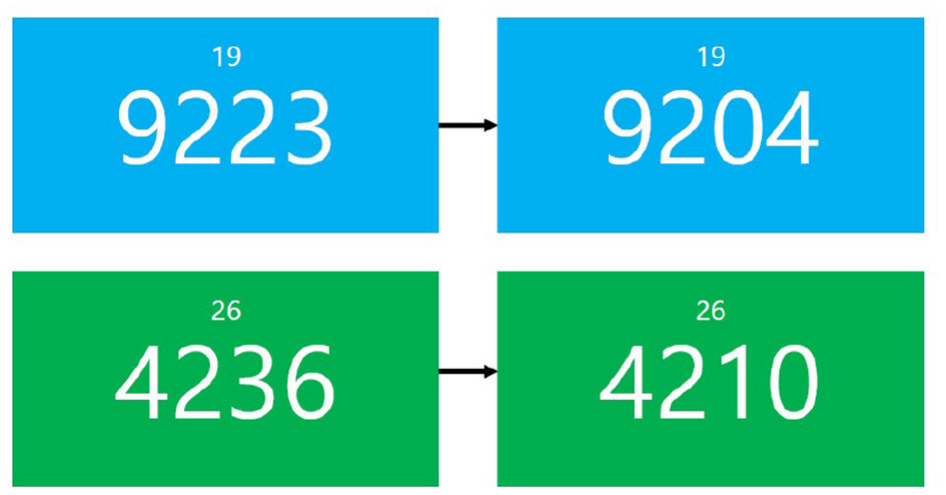
Examples of mental arithmetic task. Background color changes when the initial 4-digit number changed.

Three Experiment phases consist of “Mental arithmetic (MA) task—Survey—Interaction task—Survey.” MA task is well-known to induce mild stress (21). In this study, subjects were asked to guess the correct answer by looking at a randomly generated 4-digit number in the center of a screen and subtracting a constant 2-digit number appearing above the 4-digit number as shown in the Figure 5. After subjects said aloud their answer, they could see whether their answer was correct or not by looking at the 4-digit number that followed. The four- and two-digit numbers used for subtraction were renewed every 30 mental arithmetic problems.

As per the hypothesis, Interaction task presents one among three types of MR content: observing the movement of a non-reactive virtual cat (referred to as “Observation”), interacting with a virtual cat whose responses can be seen (“Visual”), and both seen and heard (“Audiovisual”). The duration of each Interaction task was set to 3 min, the average time for all defined commands to be repeated up to two times through the pilot test. To compare the effect of adding or reducing the virtual cat's response, the Interaction task presents three MR contents in two orders: “Observation—Visual—Audiovisual” and “Audiovisual—Visual— Observation.”

After each Experiment phase is completed, the 5-min Rest is followed. As a result of several pilot tests, the overall HRV values returned to the baseline when a 5-min period was given. In order to remove the influence of the previous tasks, subjects were allowed to take a rest and sit comfortably for 5 min.

### 4.3. Electrocardiogram recording

At the Preparation phase, a wireless ECG sensor was attached to record ECG continuously until the end of the experiment. All subjects had to wear a patch-type electrocardiogram sensor (T-REX, Taewoong Medical, Korea) on the proper location to measure lead II ECG. The sensor is rectangular-shaped with removable patch that can non-invasively assess the electrical activity of the heart.

After the recording, mean HR was calculated. In this study, We specifically focused on the HR, which can intuitively understand that the value changes according to the state of tension and rest. An increase in HR suggests a state of tension, i.e., being under stress, whereas HR decreases during recovery (22).

### 4.4. Survey

The PANAS (Positive Affect and Negative Affect Schedule) is a clinically used questionnaire that evaluates positive and negative emotions in humans. Using a total of 20 items, including 10 items of positive emotion and 10 items of negative emotion, it has been widely used to diagnose one's emotion and mood (23). Subjects were asked to respond on a 5-point Likert scale. The higher the score, the higher the corresponding emotion. In order to prevent the subjects from knowing the purpose of this study and answering intentionally while doing MR content after the MA task, the order of negative and positive questions in the PANAS questionnaire used in the experiment was jumbled, and the survey was quick to respond as soon as each task was completed.

In addition, a question was added to the subjects to choose their current stress level from a number between 0 and 4. The higher the number, the higher the stress level.

## 5. Results

### 5.1. Average heart rate

**Figure 6:**
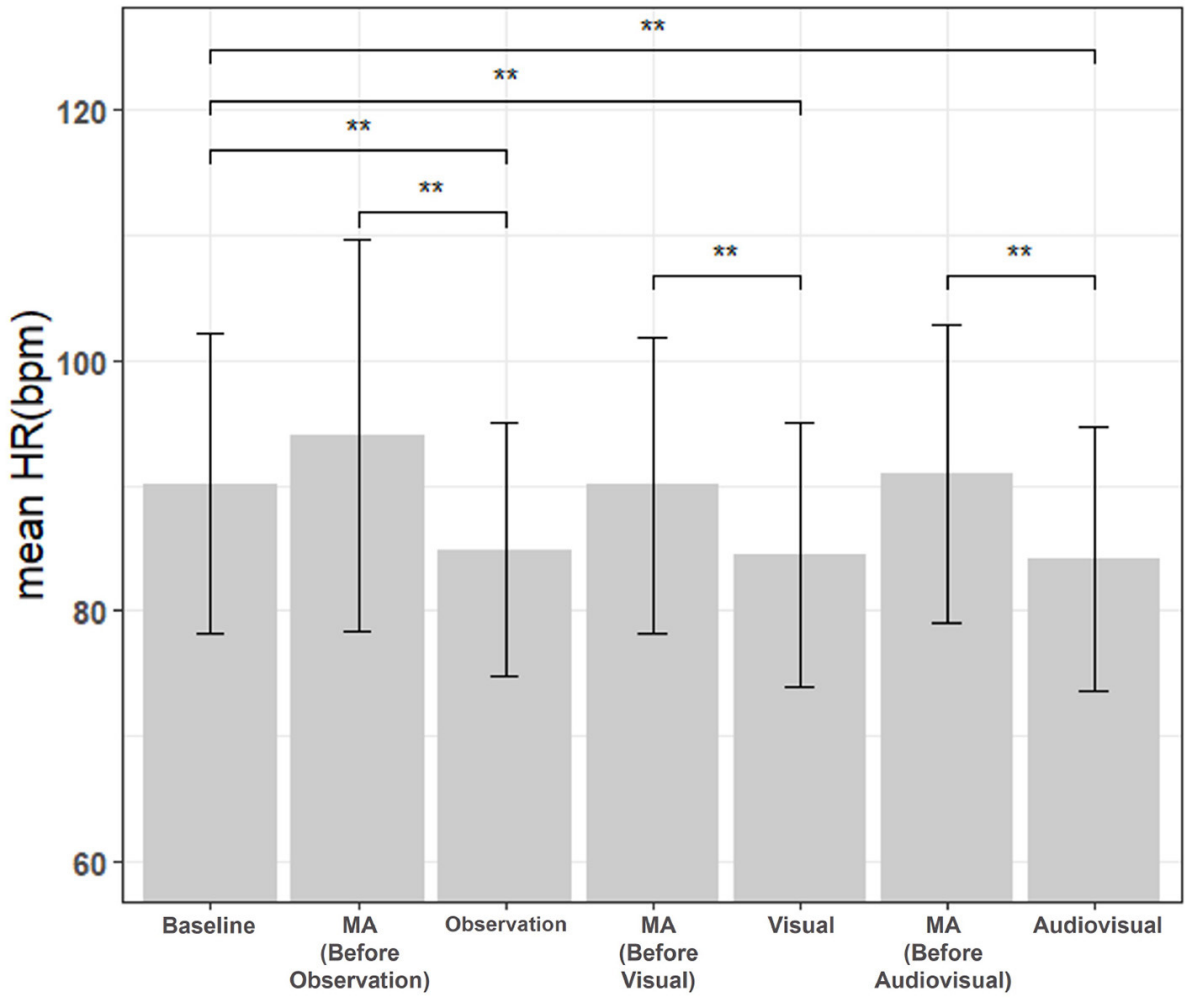
Comparison of mean HR between baseline, MA, and interaction tasks (observation, visual, and audiovisual). *N* = 30, mean±standard error, **p < 0.01.

To quantitatively evaluate the physiological response of the subject to stress during this experiment, the mean HR was analyzed. In this experiment, 30 samples were used, and the Friedman Test was performed on seven task scores, along with a post-test, since the normality was not satisfied. For comparisons between many groups over multiple data sets, Friedman test with *post-hoc* Nemenyi test was recommended in a previous study (24). Therefore, in this study, the Nemenyi *post-hoc* test was performed if Friedman's test was significant. Firstly, as shown in Figure 6, the mean HR of the three types of MR content was significantly lower compared to the mean HR of the previous MA. Also, the mean HR of MR content was significantly lower than baseline. In particular, the mean HR of “Audiovisual” was the lowest, and the statistical significance of “Audiovisual” compared to MA and baseline were also the greatest compared to those of the rest Interaction tasks, i.e., “Observation” and “Visual.”

### 5.2. Survey

**Figure 7:**
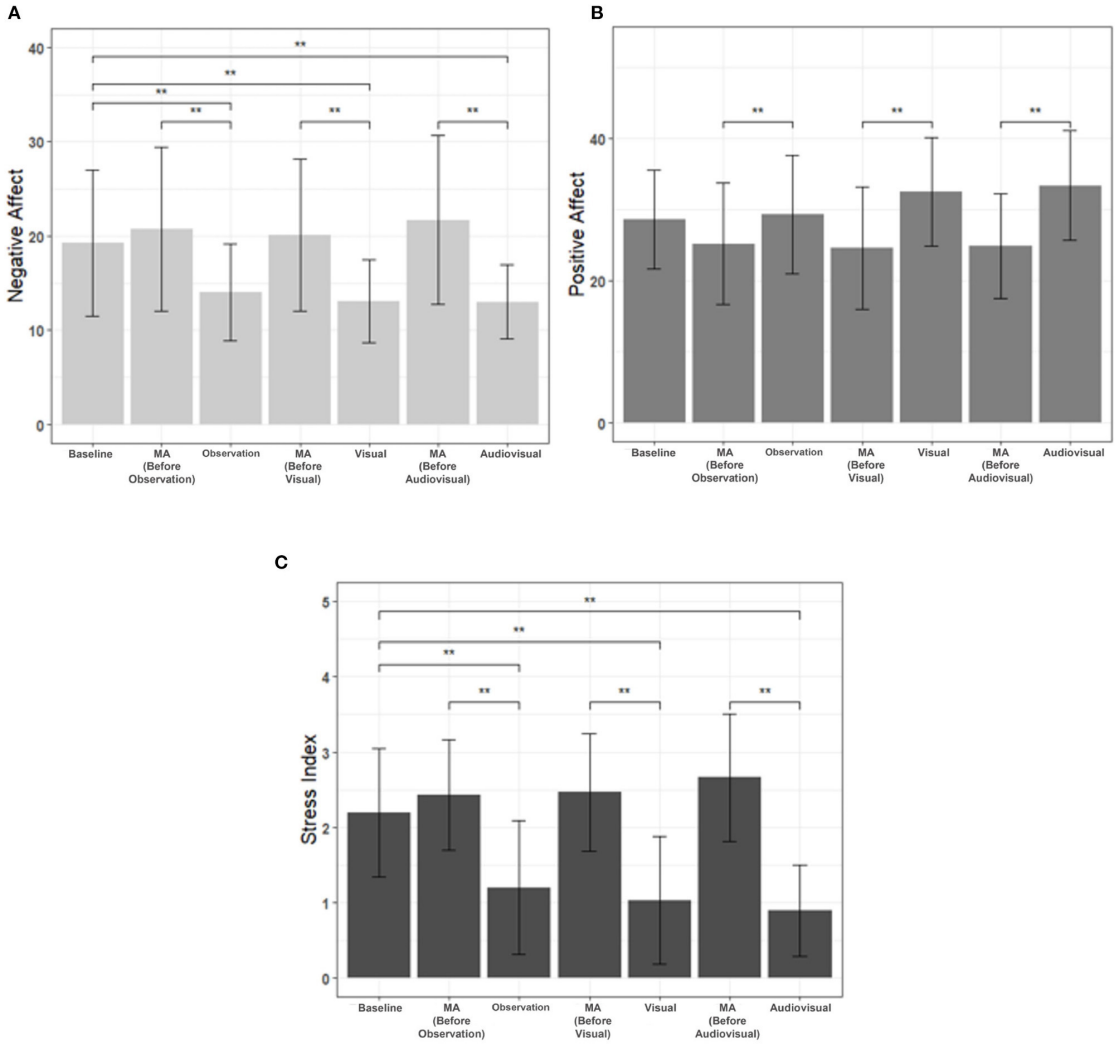
Comparison of survey results between baseline, MA, and interaction tasks (observation, visual, and audiovisual). *N* = 30, mean±standard error, *p < 0.05 and **p < 0.01. **(A)** Negative affect, **(B)** positive affect, and **(C)** stress index.

The mood status was evaluated by scoring two sub-areas of the PANAS questionnaire immediately after each task. Friedman and Nemenyi post-tests were included in the psychological evaluation for each of seven scores. In the case of negative emotions, after the three MR contents, the negative emotions scores were significantly reduced compared to those of MA. Also, they were all significantly lower than the baseline negative scores (Figure 7A). In particular, “Audiovisual” had the lowest negative emotional score, and the greatest statistical significance with baseline and MA. Next, in the case of positive emotions, the scores of the three MR contents were significantly higher than the MA scores performed immediately before, and were higher in the order of “Audiovisual,” “Visual,” and “Observation” (Figure 7B). Lastly, in the case of stress index, similar to the negative emotion score of PANAS, the three MR contents had significantly lower stress index than baseline and MA (Figure 7C). Also, “Audiovisual” had the greatest statistical significance with baseline and MA. There were no significant differences in the correction rates among three MA tasks.

### 5.3. Comparison by personal experience of MR and companion animals

To investigate the effect of personal experience with companion animals and a virtual environment, prior questions were asked to each subject before the experiment. Based on their answers, we divided subjects into two groups for each experience. The experiment was conducted by adjusting the number of assignments to each group evenly. A significant difference in mean HR was not found between groups, but it was confirmed that the mean HR of the group with prior experience of MR was lower. The subject group that had companion animals had a lower mean HR than the group that did not. This means that the subjects who have experienced MR content or have had companion animals experienced a more relaxed state on average. In the case of PANAS, the positive affect score was higher and the negative affect score was lower in the group without prior experience of MR and the group with experience raising companion animals. This result may imply that positive emotions have arisen under the influence of the novelty of experiencing MR content and familiarity with animals. Finally, in the case of stress index, the group that had companion animals was significantly lower than the group that did not. To sum up, the more you have experience raising companion animals, the greater the effect of relieving mental stress by MR content. In addition, tension was relieved more when having prior experience of the virtual environment, and positive emotions increased more when there was no prior experience of the virtual environment.

## 6. Discussion

This study aims to investigate the effect of stress relief using the MR-based human and virtual cat interaction. A total of 30 healthy female college students participated in the experiment. Experimentally, subjects were asked to do a mental arithmetic task to induce their mental stress. Subsequently, three types of MR content were compared corresponding to stress reduction: observation of the movement of the virtual cat (Observation), visual interaction with the virtual cat (Visual), and audiovisual interaction with the virtual cat (Audiovisual). For quantitative evaluation of the stress-reduction effect, a single-lead ECG and a questionnaire survey were used.

Firstly, mean HR showed significant changes due to stress and recovery. Mean HR was significantly decreased in all three types of MR when compared to MA. These results are consistent with the general findings of low HR during recovery in the ECG study (25). Moreover, mean HRs of the three types of MR contents were significantly decreased even when compared to baseline, indicating effective stress relief. In particular, “Audiovisual” had the lowest mean HR compared to two other MR contents, “Observation,” and “Visual,” and had the greatest statistical significance with baseline and MA. In addition, it was investigated whether there was an effect on the order of the MR content. The mean HR was compared by dividing the group into a sequential group (Observation—Visual—Audiovisual) and a reverse group (Audiovisual—Visual—Observation), but the order of the MR contents did not significantly affect the effect of stress relief. Consequently, MR content employing a virtual cat can effectively relieve tension by reducing heart rate in stressful situations, and audiovisual interactions have been observed to further decrease heart rate.

Next, positive and negative affect values were compared through PANAS. Negative affect and stress index, which indicate negative emotions, were significantly decreased in all three types of MR contents compared to MA, which is the baseline and stressful situation. This suggests that MR-based HAI can help to relieve negative emotions. The positive affect was significantly higher in MR contents than in the MA, suggesting that MR-based HAI can help to induce positive emotions to the subjects. In addition, in the case of “Audiovisual,” which had an audiovisual interaction with a virtual cat, it was significantly higher than that of MA. Also, MR contents with interactions (Visual and Audiovisual) had lower negative emotional scores and higher positive emotional scores than just observing the virtual cat (Observation). Consequently, MR-based animal contents can be used to reduce mental stress and induce positive emotions, and the effect can be even greater if interaction is included.

Mental stress and relief can be influenced by a number of factors, including gender, age, occupation, personal experiences with pets, and personal experiences in virtual environments. This study has limitations in that it only targeted female university students in their 20s to control for the effects of gender, age, and occupation. Also, the order of Interaction tasks has been investigated only two sequences, ascending and descending of interactions. In order to further emphasize the effect of visual and auditory interaction, future research will need to further develop content for various age groups and genders and prove it in a more specific way. In this study, we tried to find the most natural and high-quality model to make our users more comfortable and more interactive, so we decided that the cat model we used was the best fit. However, we plan to add another animal model such as a dog, which is the most familiar and friendly companion animal to many people, in future studies. In addition, compared to robots or mobile apps, AR and MR clearly have the advantage of sharing real physical space with virtual animals, giving a greater sense of reality, but the critical problem is that people do not generally own AR headsets. In order to be widely applied to regular treatment of mental healthcare or AVP activities in the future, further research will be essential.

## 7. Conclusion

In this study, as an alternative to interaction with real animals, interaction content was developed between humans and virtual animals using MR, a core technology of the 4th industrial revolution. Through gestures or voice commands, users can easily interact with virtual animals. In order to intensively verify the effect of interaction with virtual animals, three types of MR contents were created: content without interaction, content with interaction using visual feedback, and content with interaction using audiovisual feedback. The effect of relieving mental stress was evaluated using physiological indicators and psychological questionnaires. As a result, all three types of MR contents had the effect of reducing mental stress regardless of the presence or absence of interaction with the virtual cat and the type of interaction. However, when both visual and auditory feedback were provided, the effect on physiological response and psychological state was the greatest. The results of this study show that interaction with virtual animals can reduce the stress of the younger generation, who are most familiar with virtual environments than other generations. We expect this study can contribute to the wider application of MR in the field of mental healthcare.

## Data availability statement

The original contributions presented in the study are included in the article/supplementary material, further inquiries can be directed to the corresponding author.

## Ethics statement

The studies involving human participants were reviewed and approved by the Institutional Review Board of Sookmyung Women's University. The patients/participants provided their written informed consent to participate in this study.

## Author contributions

HN designed the methods, performed the experiments, analyzed the results, and wrote the manuscript. S-YD designed the methods, discussed the results, and extensive revisions to the paper. Both authors contributed to the article and approved the submitted version.

## Footnotes

^1^ https://www.microsoft.com/en-us/hololens

^2^ https://assetstore.unity.com/packages/3d/characters/animals/mammals/kitten-short-132026

^3^ https://mixkit.co/free-sound-effects/cat/

^4^ https://assetstore.unity.com/packages/tools/audio/game-voice-control-offline-speech-recognition-178047
